# microRNAs: Key Regulators in Plant Responses to Abiotic and Biotic Stresses via Endogenous and Cross-Kingdom Mechanisms

**DOI:** 10.3390/ijms25021154

**Published:** 2024-01-18

**Authors:** Tianze Ding, Wenkang Li, Fuguang Li, Maozhi Ren, Wenjing Wang

**Affiliations:** 1 Zhengzhou Research Base, National Key Laboratory of Cotton Bio-Breeding and Integrated Utilization, School of Agricultural Sciences, Zhengzhou University, Zhengzhou 450001, China; qdjndtz@163.com (T.D.); liwenkang980122@163.com (W.L.); aylifug@caas.cn (F.L.); 2 Institute of Cotton Research, Chinese Academy of Agricultural Sciences, Anyang 455000, China

**Keywords:** plant-derived miRNAs, cross-kingdom regulation, exosome-like nanovesicles, synthetic biology

## Abstract

Dramatic shifts in global climate have intensified abiotic and biotic stress faced by plants. Plant microRNAs (miRNAs)—20–24 nucleotide non-coding RNA molecules—form a key regulatory system of plant gene expression; playing crucial roles in plant growth; development; and defense against abiotic and biotic stress. Moreover, they participate in cross-kingdom communication. This communication encompasses interactions with other plants, microorganisms, and insect species, collectively exerting a profound influence on the agronomic traits of crops. This article comprehensively reviews the biosynthesis of plant miRNAs and explores their impact on plant growth, development, and stress resistance through endogenous, non-transboundary mechanisms. Furthermore, this review delves into the cross-kingdom regulatory effects of plant miRNAs on plants, microorganisms, and pests. It proceeds to specifically discuss the design and modification strategies for artificial miRNAs (amiRNAs), as well as the protection and transport of miRNAs by exosome-like nanovesicles (ELNVs), expanding the potential applications of plant miRNAs in crop breeding. Finally, the current limitations associated with harnessing plant miRNAs are addressed, and the utilization of synthetic biology is proposed to facilitate the heterologous expression and large-scale production of miRNAs. This novel approach suggests a plant-based solution to address future biosafety concerns in agriculture.

## 1. Introduction

MicroRNAs (miRNAs) are a class of small, non-coding RNAs widely found in animals and plants. They are crucial in regulating gene expression by targeting and binding mRNAs, resulting in their subsequent silencing or degradation. Accordingly, miRNAs participate in myriad critical processes, including cellular differentiation, proliferation, and survival [1]. Although miRNAs are typically only 22–24 nucleotides (nt), they are among the most abundant gene-regulating molecules, affecting various protein-encoding genes [2]. In 1993, the first miRNA, *lin-4*, was identified in the nematode *Caenorhabditis elegans*. Specifically, *lin-4* regulates the timing of postembryonic developmental events in *C. elegans* by negatively regulating the expression of the nuclear protein *lin-14* [3]. Seven years later, a second miRNA, *let-7*, was discovered in *C. elegans* [4]. Similar to *lin-4*, *let-7* regulates nematode development by binding to the 3′-untranslated region (UTR) of specific target mRNAs, including *lin-14*, *lin-28*, *lin-41*, *lin-42*, and *daf-12*. Knocking out *lin-4* or *let-7* in *C. elegans* results in severe developmental abnormalities, suggesting they are crucial in organismal development [5]. These findings have sparked widespread interest in exploring the functions of other miRNAs. Notably, the most commonly used miRNA database, miRBase version 22.1 (accessed on 26 October 2023; https://www.mirbase.org), has registered 38,589 miRNAs from 271 organisms [6], with most existing as single copies, multiple copies, or clusters [7,8,9].

miRNAs are processed from transcripts with hairpin structures produced endogenously within cells [10]. Animal miRNAs are a class of evolutionarily conserved genes that are key regulators of cellular activity [11] and serve as biomarkers for detecting the state of in vivo microenvironments, including analysis of the tumor microenvironment [12,13,14,15,16]. However, plant and animal miRNAs have evolved independently and, therefore, differ in their corresponding sequences, precursors, and maturation processes [17,18]. The regulatory effects of plant miRNAs on endogenous genes have significant impacts on crop growth and development, playing important roles in resistance to abiotic stress, such as drought, cold, and nutrient deficiencies, as well as biotic stress, such as pathogens and harmful pests.

miRNA-dependent cross-species regulatory mechanisms of plant disease resistance have been reported. For example, Zhang et al. found that endogenous miRNAs of cotton, namely miR166 and miR159, translocate into pathogenic bacteria, regulating their pathogenicity and, ultimately, inhibiting *Verticillium dahliae* colonization of cotton, thus improving cotton resistance to bacterial infection [19]. Furthermore, Chen et al. found that miR162a in rice can negatively regulate brown planthopper reproduction and development via transboundary silencing, representing an important component of the rice defense system against brown planthopper infestation [20]. The cross-species regulatory mechanisms of plant miRNAs can provide novel strategies for crop genetic breeding and novel pesticide development.

This article comprehensively reviews miRNA biogenesis and regulatory mechanisms. It delves into how miRNAs respond to biotic and abiotic stress, employing endogenous and cross-kingdom regulatory mechanisms. Furthermore, it presents the most recent insights into the functional characteristics of miRNAs as potential regulators in the stress responses of plants. Finally, the prospects and challenges of miRNA applications are presented, suggesting potentially viable solutions.

## 2. Plant miRNA–Associated Biosynthesis and Plant Physiology

### 2.1. Biosynthesis of Plant miRNAs

miRNAs exert central roles in cell differentiation, proliferation, and survival by binding to complementary target mRNAs, resulting in their translational repression or degradation [1]. Plant miRNAs are produced through a multistep process encompassing the transcription, precursor processing, methylation, and assembly of miRNA-induced silencing complexes (miRISC) [21]. Based on sequence complementarity, miRNAs directly impact mRNA cleavage, translational repression, and DNA methylation [21]. Importantly, miRNA production is typically conserved across various species [1,22].

**Figure 1:**
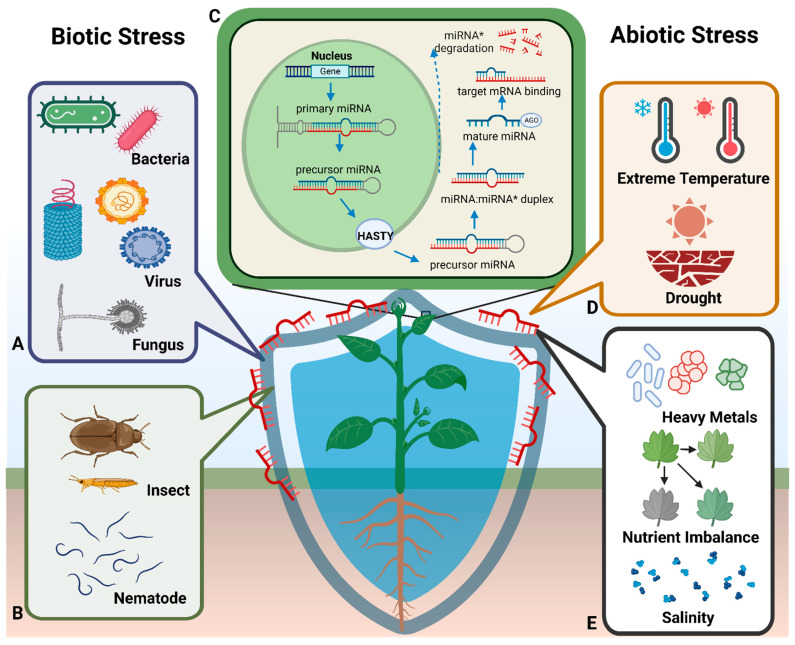
miRNA synthesis in plants and associated plant responses to abiotic and biotic stress. (**C**) Summary of the miRNA synthesis process in plants: pri-miRNAs are translated in the nucleus, processed into pre-miRNAs by the DCL1 enzyme, and transported to the cytoplasm via HASTY, where mature miRNAs are generated. (**A**,**B**) Associated biotic stress (e.g., microorganisms and insects). (**D**,**E**) Associated abiotic stress (e.g., extreme temperature, drought, heavy metal stress, nutrient stress, and salt stress).

In animals, the processing of primary miRNA (pri-miRNA) occurs in the nucleus and cytoplasm via two distinct endonucleases (Drosha and Dicer, respectively) belonging to different subclasses of the RNase III family [23,24]. Pri-miRNAs produce 60–70 nt precursor miRNAs (pre-miRNA) after cleavage [25], which undergo cleavage mediated by the nuclear class II RNase III Drosha-DGCR8 complex and are delivered to the cytoplasm through exportin-5. In the cytoplasm, the pre-miRNA molecules undergo further cleavage by the class III RNase III enzyme Dicer, creating an ~22-nt miRNA duplex [1,26]. In contrast, both stages of pri-miRNA processing in plants are catalyzed within the nucleus by Dicer-LIKE 1 (DCL1), a Dicer homolog [21,27,28]. Plant miRNA genes are predominantly located in intergenic regions of the genome and function as independent transcriptional units. Although miRNA promoters are structurally similar to those of protein-coding genes [29], a small number of miRNAs located in the intronic sequences of protein-coding genes can be co-transcribed with their host genes [30,31,32]. Genes encoding miRNAs are transcribed in the nucleus under the action of RNA polymerase II, forming the pri-miRNA, approximately several hundred nucleotides in length (Figure 1) [33,34]. Subsequently, the Dicer-like enzyme, DCL1, produces the pre-miRNA and continues to process it, forming a double-stranded miRNA [21]. The final double-stranded miRNA undergoes methylation modification at the last nucleotide of the 3′ end on both strands [35], facilitated by miRNA methyltransferase HEN1 [22,36]. The double-stranded miRNA and its complementary strand (i.e., miRNA*) become integrated into the Argonaute protein in the RNA-induced silencing complex (RISC), enabling the complex to search for and bind to the target mRNA sequence, whereas miRNA* would normally degrade [26,37]. Mature miRNAs are subsequently transported to the cytoplasm by HASTY—an exportin5 homolog—or transported to the cytoplasm by HASTY before binding to ribosomal proteins [36]. RISC silences gene expression via one of three mechanisms: (i) interfering at the protein translation level; (ii) degrading mRNA at the transcription level; or (iii) heterochromatin formation or DNA elimination at the genome level [37].

### 2.2. Role of Plant miRNAs in Plant Physiology

Plant miRNAs play important regulatory roles in various biological processes, including plant development, metabolism, responses to abiotic stress, and defense against pathogens. These miRNAs can effectively recalibrate internal plant signaling and metabolic pathways, regulate gene expression, and modulate gene responses [38]. Additionally, plant miRNAs can inhibit gene expression in pathogens, most notably regarding resistance traits, thus increasing the ability of plants to resist infection. Plant miRNAs can also control the adaptation of cellular biological responses to environmental stress and low nutrient supplies [39,40]. Moreover, as important environmental response regulators, miRNAs regulate gene expression and mediate plant growth and development under low-temperatures, heat stress, salt stress, and fungal stress [38].

As the study of miRNAs advances, an increasing body of evidence has demonstrated their roles in regulating plant cell differentiation, embryo formation, fertility, plant flowering, and fruiting, along with regulating plant type and seed yield. For example, Luo et al. reported that miR397 is highly expressed in undifferentiated rice embryonic healing tissues, leading to the downregulation of laccase genes; this causes the formation of thin-walled meristematic cells [41]. Alternatively, Hou et al. found that overexpressing miR171 in tomatoes results in larger tomato plants by suppressing the target gene family GRAS (SLGRAS24) [42]. Similarly, rice miR397 increases seed size and promotes spike branching by downregulating *OsLAC* expression, improving rice yield [43]. Moreover, *Arabidopsis* plants overexpressing miR397 can produce more inflorescence buds, increasing seed yield [44]. Meanwhile, miR528 participates in regulating the tillering number and regeneration ability of switchgrass through the miR528-SOD module, which enriches the regulatory network of miRNA-target genes [45].

## 3. Plant miRNAs Mediate Plant Resistance via Endogenous Regulation

Plant-derived miRNAs not only participate in regulating the host plant’s endogenous genes related to growth and development but also contribute to responses to abiotic (Table 1) and biotic (Table 2) stress by modulating the host’s endogenous target genes, thus impacting the agronomic traits of crop varieties.

### 3.1. miRNA Responses to Abiotic Stress

#### 3.1.1. miRNAs and Heavy Metal Stress

Heavy metals, such as cadmium, aluminum, arsenic, lead, and mercury, are typically absorbed by plant root systems and can significantly inhibit plant growth [46]. Cadmium is a particularly persistent trace metal and a hazardous environmental pollutant [47] that is not essential for key plant metabolic or growth processes [48]. Therefore, Ali et al. constructed *Arabidopsis thaliana* miR397 mutant lines to investigate the molecular mechanisms responsible for the potential relationship between cadmium tolerance and miR397 expression [49]. They found that overexpressing miR397 alters lignin content and reduces cadmium tolerance by regulating laccase 2, 4, and 17 expressions. Similarly, knocking down miR535 improves cadmium tolerance in rice plants under cadmium stress, while overexpressing miR35 elicits the opposite effect compared to wild-type (WT) plants [50]. Additionally, Yan et al. found that growth inhibition, oxidative damage, and antioxidant enzyme disorders are more pronounced in tomato plants overexpressing miR398 under cadmium stress than in WT plants. Conversely, miR398 down-regulation exerts a protective effect against cadmium stress in tomato plants by regulating antioxidant enzyme activities alongside cadmium uptake and translocation [51].

#### 3.1.2. miRNAs and Drought Stress

Drought is a major stressor that negatively impacts plant growth and development, leading to significant crop losses globally [52]. Notably, Jiang et al. reported that the loss of miR159 function in *A. thaliana* increases drought tolerance, attributed to fewer open stomata and lower water loss rates [53]. Meanwhile, Li et al. found that *Arabidopsis* plants overexpressing miR169a exhibit enhanced leaf water loss and greater sensitivity to drought stress than WT plants [54]. Additionally, reducing miR1119 expression in wheat roots increases MYC2 levels, upregulating the activity of certain enzymes, including catalase (CAT), peroxidase (POD), and superoxide dismutase (SOD), effectively improving drought resistance [55].

#### 3.1.3. miRNAs and Temperature Stress

Extreme temperatures can disrupt normal cellular and biochemical functions in plants, inhibiting their normal growth. High-temperature stress leads to oxidative damage through the generation of reactive oxygen species (ROS), which damage organelles by disrupting membranes via lipid peroxidation, ultimately reducing chlorophyll content and diminishing photosynthetic capacity [56,57]. Alternatively, low temperatures adversely affect plant growth and yield, significantly reducing growth efficiency [58]. Indeed, some plants can be irreversibly damaged by low-temperature stress [59]. To address these challenges, Matthews et al. found that miR156 can help alleviate high-temperature stress in alfalfa; miR156-overexpressing plants exhibit notably enhanced antioxidant scavenging capacity, reducing ROS-associated damage [60]. Moreover, using high-throughput screening, Zeng et al. reported that miR166e, miR319, and Bra-novel-miR3936-5p may have important roles in plant responses to cold stress [61]. This was further demonstrated by Shi et al., who constructed sha-miR319d-overexpressing tomato plants with enhanced cold and heat stress tolerance [62].

#### 3.1.4. miRNAs and Nutrient Stress

A disparity exists between the nutritional requirements of plants in agricultural settings and those in their natural environments; nonetheless, irrespective of the environment, deficiencies in essential elements can induce nutritional stress, which can negatively affect normal plant growth and development [63]. miR156 expression becomes upregulated in response to nitrogen deficiency, promoting plant height development by regulating SQUAMOSA promoter-binding protein-like (SPL) genes [64] and primary and lateral root growth by regulating the expression of *NAC4*, *ARF2*, and *AFB3* [65]. Alternatively, Xu et al. analyzed the transcriptome of soybean roots and leaves under phosphorus sufficiency and deficiency. They established that miR169, miR395, miR397, and miR399 are regulated to alleviate phosphorus deficiency-induced stress [66]. Moreover, under potassium deficiency, miR171 and miR166 expression levels become markedly altered in wheat; wheat seedlings synthesize more miRNAs, resulting in increased potassium absorption during the pre-growth phase to promote root differentiation and development [67].

#### 3.1.5. miRNAs and Salt Stress

Extended exposure to saline conditions can lead to ROS production [68], which can cause oxidative damage to cellular components, including proteins, lipids, and DNA, disrupting critical plant biochemical processes [69,70]. The resultant tissue damage can lead to a reversible slowing of metabolism and growth and, in more severe cases, irreversible cell death [71]. Under salt stress, the expression levels of nine representative miRNAs, including miR159, miR168, miR169, miR172, miR393, miR395, miR396, miR399, and miR408, vary in different plant species [72]. Interestingly, the same miRNAs may have opposite expression patterns in different plant species and fulfill differing roles under salt stress. For example, in tobacco, miR396 overexpression can improve water retention and proline content, helping reduce ROS production and improve salt resistance [73]. In contrast, overexpression of miR396 reduces salinity stress tolerance in rice and *A. thaliana* [74].

**Table 1:** Summary of endogenous miRNA regulation in plants under abiotic stress.

Plant	miRNA Regulation	Function	Target Gene(s)	Reference(s)
*Arabidopsis thaliana*	miR397	Cadmium tolerance	*LAC2/4/17*	[49]
Rice	mir535		*SPL7*	[50]
Tomato	miR398		*IRT1*, *IRT2NRAMP2*, *HMA3*	[51]
Barley	miR156		*HvNAT2*	[75]
*Arabidopsis thaliana*	miR159	Drought	*ABI5*	[53]
*Arabidopsis thaliana*	miR169a		*NFYA5*	[54]
Wheat	miR1119		*MYC2*	[55]
*Arabidopsis and rice*	miR393		*AsAFB2*, *AsTIR1*	[76]
Alfalfa	miR156	Temperature stress	*SPL*	[60]
Rapeseed	miR166e, miR319, miR3936-5p		–	[61]
Tomato	sha-miR319d		*MYB83*, *CBF1*, *HSFA1A*, *HSFA1B*, *HSP90*, *ZAT12*, *ZAT10*	[62]
Tea plants	miR156	Nutritional stress	*SPL*, *DFR*	[64]
Peanut	miR156		*NAC4*, *ARF2*, *AFB3*	[65]
Soybean	miR169, miR395, miR397, miR399		–	[66]
Wheat	miR171, miR166		*ARF*	[67]
Tomato	miR167a	Salt stress	*DREB2A*	[77]
Tobacco	miR396a		*NtGRF1*, *NtGRF3*, *NtGRF7*, *NtGRF8*	[73]
Rice	miR396c		*ABRE*	[74]

### 3.2. miRNA Responses to Biotic Stress

In natural environments, plants are frequently exposed to biotic stress from various sources, including bacteria, fungi, viruses, and pests. Extensive research has shown that miRNAs are effective regulators of plant resistance to pathogen infections [40,78]. When a host plant is attacked by pathogens, miRNA expression responds rapidly, altering the expression of its downstream target genes; ultimately, this regulates the plant’s resistance to pathogenic infection by affecting processes such as hormone transduction, disease resistance gene expression, and secondary metabolism (Table 2).

#### 3.2.1. miRNAs Mediate Pathogen Resistance in Plants through Hormonal Signaling Pathways

Zhao et al. observed that tomato miR319-TCP4 plays a key role in influencing plant resistance to root-knot nematodes by regulating the expression of genes related to jasmonic acid (JA) synthesis and altering the levels of endogenous JA in leaves [79]. Moreover, Pinweha et al. found that, when cassava is infested with *Colletotrichum gloeosporioides* f. sp., the expression of miR393 and miR160 promotes a defense response against anthracnose by down-regulating the target genes *TIR1* and *ARF*, thus suppressing growth hormone signaling [80]. Similarly, when *Arabidopsis* is exposed to *Pseudomonas syringae* flagellin, miR393 expression is induced, leading to the negative regulation of the F-box growth hormone receptor *TIR1*. This inhibition suppresses growth hormone signaling, promoting antimicrobial resistance in *A. thaliana* [81]. Furthermore, infestation of *Malus hupehensis* with *Botryosphaeria dothidea* promotes the upregulation of host miR168, targeting MhAGO1 inhibition and facilitating salicylic acid (SA)-mediated defense responses. These defense responses can delay the symptom progression of pathogen-inoculated leaves and positively regulate resistance to pathogenic bacteria [82]. Additionally, Zhang et al. reported the involvement of miR394 in the negative regulation of biotic stress. Notably, miR394 overexpression in tomato plants inhibits the expression of its target gene, *LCR*; this, in turn, inhibits the expression of JA synthesis–related genes, reducing resistance to disease-causing mildew [83]. Finally, Yang et al. reported that, compared to WT poplar trees, miR159a overexpression in transgenic poplars can enhance disease resistance following inoculation with the necrotrophic fungus *C. chrysosperma*. These findings demonstrate that JA/ethylene (Et)-inducible signaling is involved in the dynamic response to biotic stress in WT and miR159a-overexpressing lines [84].

Citrus can resist *Candidatus Liberibacter asiaticus* (CLas) by activating the miR2977–*SAMT* cascade reaction, which further utilizes methyl salicylate (MeSA) to regulate phytosome defense [85]. Interestingly, CLas hijacks key host processes by manipulating critical miRNAs in citrus, while host miRNAs also coordinate the regulation of defense-related genes. Liu et al. performed differential gene analysis of resistant *Cladosporium fulvum* plants and identified 54 miRNAs that may regulate hormone signaling [86]. The downregulation of miR9472 potentially upregulates the expression of SAR deficient 1 (*SARD1*), a key regulator of ICS1 (isochorismate synthase 1) induction and SA synthesis, to improve the level of SA in resistant plants. These findings provide a more comprehensive gene circuit and valuable targets for modulating resistance to pathogens. Additionally, silencing miR530 enhances plant defense against *Verticillium dahliae*, whereas its overexpression weakens plant resistance [87]. This is related to the function of miR530, which degrades *GhSAP6* mRNA. Knockdown of *GhSAP6* also reduces plant resistance due to the down-regulation of SA-related gene expression, including *GhNPR1* and *GhPR1*.

#### 3.2.2. miRNAs Mediate Pathogen Resistance in Plants by Regulating Plant Disease Resistance Proteins

The miRNA response to plant disease resistance primarily functions as a regulatory mechanism that targets plant disease resistance proteins, such as NB-LRR, chitin, and lectin. Plant NB-LRR proteins can initiate plant defense responses following microbial infection. In tomato plants, negative regulation of NB-LRR proteins by miR482 and miR5300 in vivo reduces resistance to *Fusarium oxysporum* f. sp. *lycopersici* infection [88]. In *A. thaliana*, the in vivo downregulation of miR393 and upregulation of its target genes, lectin receptor-like kinases (LecRLKs), occurs after treatment with bacterial lipopolysaccharide (LPS); ultimately, this response enhances the basal resistance of *A. thaliana* to LPS [89]. Zhang et al. reported that, in apple leaves inoculated with leaf spot fungus, MdmiR395 expression increases while that of its target genes, *MdMRKY26* and *MdWRKYN1*, decreases; moreover, overexpression of *MdWRKY* or inhibition of MdmiR395 increases the expression of pathogenesis-related genes, enhancing the apple tree’s resistance [90]. Furthermore, using small RNA sequencing, Li et al. identified 58 miRNAs associated with potato virus A (PVA) infection [91]. miR482d-3p, miR397-5p, and other miRNAs become down-regulated, increasing PR gene expression. This study provides transcriptome-wide insights into the molecular basis of PVA resistance in potato leaves.

Leucine-rich repeat proteins (NLRs) are critical to the plant–pathogen defense response, while 22 nt miRNAs are essential for NLR regulation, improving the response rate of plants to most pathogens [92]. NLRs are the largest class of disease-resistance (R) proteins in plants [93]. miR2118 in rice negatively regulates *Xanthomonas oryzae pv. oryzae* resistance via negative regulation of three nucleotide-binding NLR genes (*LOC_Os08g42700.1*, *LOC_Os01g05600.1*, and *LOC_Os12g37290.1*) [94].

#### 3.2.3. miRNAs Regulate Reactive Oxygen Species Accumulation and Plant Secondary Metabolism Responses to Abiotic Stress

When rice plants are subjected to viral damage, miR528 expression is suppressed, promoting the expression of its target gene, L-ascorbate oxidase (*AO*), and, subsequently, reducing AO-mediated ROS accumulation [95]. Moreover, the miR528-AO defense module is regulated by SPL9, which binds to specific motifs within the miR528 promoter region, activating miR528 gene expression [96]. Similarly, reducing miR398b expression in tomato plants reduces ROS accumulation and up-regulates MeJA-responsive defense genes, increasing plant resistance to *Botrytis cinerea* [97]. Downregulation of miR397 expression during the plant response to pathogenic microbial infestation enhances laccase activity, induces the synthesis of lignin and other phenolic polymers, and causes rapid deposition in the cell wall; ultimately, this limits the growth and invasion of pathogenic microorganisms [98,99]. For example, infection of potatoes with PVA and maize with sugarcane mosaic virus (SCMV) is inhibited by the downregulation of miR397 expression, enhancing laccase activity and increasing lignin formation [91,100].

Alternatively, Chandran et al. demonstrated that transgenic rice lines overexpressing miR396 are highly susceptible to *Aspergillus oryzae* infection, whereas those overexpressing its target genes (*OsGRF6*, *OsGRF7*, *OsGRF8*, and *OsGRF9*) exhibit enhanced *A. oryzae* resistance [101]. Furthermore, miR396 and GRF8-OE plants exhibit increased flavonoid content, which is associated with enhanced pathogen resistance, revealing a novel pathogen resistance mechanism mediated by the miR396–GRF8–F3H–flavonoid pathway [102]. In contrast, overexpression of miR396 in alfalfa enhances resistance to *Spodoptera litura* larvae; this may be attributed to the increased lignin content and enhanced biosynthesis of low-molecular-weight flavonoids and glucosinolates [103].

**Table 2:** Summary of endogenous miRNA regulation in plants under biotic stress.

Plant	miRNA Regulation	Pathogen	Pathogen Resistance Function	Target Gene		Reference(s)
Cassava	miR393, miR160	*Colletotrichum gloeosporioides f.* sp.	Hormonal signaling	IAA	TIR1 ARF	[80]
*Arabidopsis thaliana*	miR393	*Pseudomonas syringae*			TIR1	[81]
*Malus hupehensis*	miR168	*Botrytis cinerea*		SA	MhAGO1	[82]
Tomato	miR319	Root-knot nematode		JA	TCP4	[79]
Tomato	miR394	Disease-causing mildew			LCR	[83]
Poplar tree	miR159a	Necrotrophic fungus *C. chrysosperma*		JA\ET	ERF, PR3, MPK3, MPK6	[84]
Tomato	miR482, miR5300	*Fusarium oxysporum f.* sp. *lycopersici*	Plant disease resistance proteins	NB-LRR		[88]
*Arabidopsis thaliana*	miR393	Bacterial lipopolysaccharide		LecRLKs		[89]
Apple	miR395	Leaf spot fungus		PR		[90]
Rice	miR528	Rice stripe virus	Reactive oxygen and plant secondary metabolism	AO, SPL9		[95,96]
*Malus hupehensis*	miR397b	*Botryosphaeria dothidea*		Laccase		[98,99]
Potato	miR482d, miR397	Potato virus A				[91,100]
Corn	miR168, miR528, miR159, miR397, miR827	Sugarcane mosaic virus				
Rice	miR396	*Aspergillus oryzae*		Flavonoids		[101,102]
Alfala	miR396	*Spodoptera litura* larvae		Lignin, Flavonoids, Glucosinolates		[103]

## 4. Plant miRNAs Mediate Plant Resistance via Cross-Kingdom Regulation

In addition to their role in regulating the expression of endogenous genes, miRNAs also function as intercellular signaling molecules, facilitating cross-species regulation between plants and other plants, microorganisms, and animals. In 2012, Zhang et al. determined that miR168a—a plant-derived miRNA—can enter the circulatory system of animals through food intake. In mice, this miRNA targets and inhibits the expression of low-density lipoprotein receptor adapter protein 1 (LDLRAP1), resulting in the cross-kingdom regulation of lipid metabolism [104]. Ultimately, this study provided a foundation for research into miRNA-mediated cross-species regulation by plants.

A study by Chen et al. found that plant miRNAs in bee diets have an inhibitory effect on the ovary and overall growth and development of worker honeybee larvae. In contrast, royal jelly does not contain plant miRNAs; therefore, young bees that consume royal jelly develop into queen bees [105]. This finding challenges the previous understanding that royal jelly contains specific beneficial substances that allow young bees to develop into queens. Moreover, plant miRNAs can fine-tune bee caste development, providing insights into the interactions and co-evolution across different biological kingdoms.

In a study by Liu et al., next-generation sequencing identified 30 plant miRNAs in a healthy Chinese population. These sequences did not align with any established sequences within the human genome; in contrast, they aligned identically with plant miRNAs commonly found in food [106].

Hence, growing evidence indicates that plant miRNAs exhibit cross-species and cross-kingdom regulation (Table 3), serving as mediators of gene silencing and as a link between animal, plant, and microbial communities.

### 4.1. miRNA-Mediated Regulation of Plant–Plant Gene Expression

miRNAs can play related roles throughout the whole plant [107,108]. For example, miR399 and miR156 can be transported throughout the plant, serving as signal molecules [109,110,111]. In plants overexpressing miR399 and miR156 and exposed to exogenous *A. thaliana*, the expression of PHO2 and SPLs is decreased, respectively. When *A. thaliana* plants overexpressing miR399 and WT are cultured in the same liquid medium, the expression of PHO2 in WT plants decreases, while plants overexpressing miR156 inhibit the SPL expression of nearby WT plants [112].

**Figure 2:**
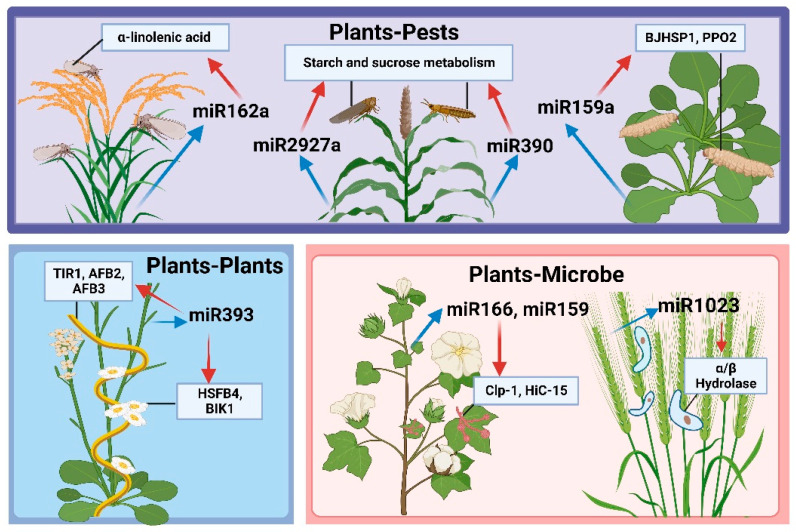
Cross-kingdom miRNA regulation.

*Cuscuta* is a genus of parasitic plants with specialized physiology. *Cuscuta* spp. employs climbing stems to attach to other plants, forming specialized structures called haustoria at the contact sites. These haustoria can be used to extract water and nutrients from the host. As shown in Figure 2, Shahid et al. reported that, during the parasitization of *A. thaliana* by *Cuscuta*, corresponding *Cuscuta* miRNAs (22 nt long) target specific *A. thaliana* mRNAs; this increases cleavage of the mRNAs, heightens production of secondary siRNAs, and reduces mRNA content [113]. Indeed, mutations in two loci encoding the target mRNAs in *A. thaliana* can significantly accelerate the growth rate of *Cuscuta* after parasitization, suggesting that increased miRNA-mediated targeting of host mRNAs can enhance *Cuscuta* parasitism. Moreover, Hudzik et al. identified several miRNAs in *Cuscuta* that target and regulate specific host mRNAs in a cross-species manner. The targeted host mRNAs in *A. thaliana* include those involved in biodefense, hormone signaling, and vascular development [114].

Finally, Betti et al. demonstrated that plants can secrete miRNAs. Notably, plants with a high miRNA secretion capacity influence neighboring plants with lower miRNA secretion by inducing post-transcriptional gene silencing. Ultimately, this confirms that miRNAs can function as signaling molecules, enabling plant-to-plant communication [112].

### 4.2. miRNA-Mediated Regulation of Plant–Microbe Gene Expression

miRNA-mediated cross-species genetic regulation is important in plant–microbe interactions. For example, Zhang et al. found that *V. dahliae* infection induces the expression of endogenous miRNAs miR166 and miR159 in cotton, which translocates to the pathogen. Ultimately, in *V. dahlia*, miR166, and miR159 inhibit the formation of micronuclei and mycelial growth via specific genetic cleavage of the pathogen virulence factors Ca^2+^-dependent cysteine protease (Clp-1) and isotrichodermin C-15 hydroxylase (HiC-15), respectively. This further inhibits *V. dahliae* colonization in cotton, enhancing cotton resistance [19].

Additionally, Meng et al. determined that miR1001 from tomatoes reduces the virulence of *Botrytis cinerea* in infected tomato plants by targeting ATP-dependent metallopeptidases and cysteine-type endopeptidases in *B. cinerea* [115]. Moreover, wheat miR1023 silences the *Fusarium graminearum* α/β hydrolase gene, hindering *F. graminearum* invasion [116]. Luo et al. identified 133 miRNAs in potatoes that are predicted targets in the genome of *Phytophthora infestans*. Upon further validation, miR0003, miR394, etc. can target multiple conserved *P. infestans* genes, providing a new approach for the control of late blight [117]. Notably, cross-kingdom regulation between plants and microbes is not limited to plants exporting specific miRNAs to silence disease-causing genes in pathogens. Pathogenic microbes can also export specific miRNAs into plants, resulting in the silencing of disease-resistant genes and accelerating infection [118,119].

### 4.3. miRNA-Mediated Regulation of Plant–Pest Gene Expression

Recently, plant miRNAs have been shown to protect against insect damage by regulating plant growth and development. In addition, some plant miRNAs enter the body of feeding insects and regulate insect growth and development, exerting a significant effect on natural plant–pest interactions [120]. For example, Chen et al. found that brown planthopper damage to rice induces the expression of rice miR162a. This initiates a defense response against infestation by down-regulating brown planthopper reproduction and development via transboundary silencing and inhibiting the α-linolenic acid metabolic pathways in rice, reducing brown planthopper host selectivity. Indeed, the JA-mediated defense response has created long-term evolutionary competition between the pest and host [20]. Alternatively, Zhang et al. identified plant-derived miRNAs in the hemolymph of the cruciferous cabbage moth, *Cechetra minor*. They reported that these miRNAs can enter the circulatory system by penetrating the midgut barrier; notably, these plant-derived miRNAs may play regulatory roles in *C. minor* [121]. Similarly, 13 sorghum and 3 barley miRNAs were detected in two cereal aphids, *Schizaphis graminum* and *Sipha flava*; the associated target genes were predicted to participate in aphid defense by inhibiting starch and sucrose metabolism [122]. Similar to the involvement of miRNAs in plant–microbe cross-species regulation, plant-derived miRNAs can regulate insect target genes; insect-derived miRNAs can enter the plant via the saliva, contributing to interspecies communication between the plant and insect [120,123].

**Table 3:** Summary of plant cross-kingdom miRNA regulation under biological stress.

Plants	miRNA-Regulation	Transboundary Species		Targets	Reference(s)
*Arabidopsis thaliana*	miR393	Plants	*Cuscuta campestris*	Maybe TIR1, AFB2, AFB3, HSFB4, BIK1	[113,114]
Terrestrial cotton	miR166, miR159	Microbe	*V. dahliae*	Clp-1, HiC-15	[19]
Tomato	miR1001		*Botrytis cinerea*	ATP-dependent metallopeptidases and cysteine-type endopeptidases	[115]
Wheat	miR1023		*Fusarium graminearum*	FGSG_03101	[116]
Rice	miR162a	Pests	Brown planthopper	α-linolenic acid metabolic pathways	[20]
*Arabidopsis thaliana*	miR159a, novel-7703-5p		Moth Cechetra minor	BJHSP1, PPO2	[121]
Sorghum and Switchgrass	miR2927a		*Schizaphis graminum*	Starch and sucrose metabolism	[122]
	miR390		*Sipha flava*		

## 5. Potential Applications and Strategies for Cross-Kingdom Plant miRNAs

There is a growing body of evidence suggesting that plant miRNAs have the ability to migrate from plants to other plant species, microbes, insects, and even mammalian cells, allowing them to regulate specific genetic processes. This property allows these small molecules to serve as alternative biopesticides in modern agriculture. As previously discussed, many of the identified plant miRNAs exhibit cross-kingdom regulatory functions. Comprehensive investigations into the mechanisms behind cross-species miRNA regulation may provide important references for the design, modification, optimization, and utilization of miRNAs.

### 5.1. Artificial miRNAs Increasing the Value of Plants

Artificial miRNA (amiRNA) technology, designed according to the principles of natural miRNA generation and action, employs a small RNA molecule that targets one or more specific genes to efficiently and specifically inhibit their expression [124]. RNA interference (RNAi) is a powerful tool for studying gene function, with siRNA and short hairpin RNAs (shRNAs) the preferred choice for the transient knockdown of gene expression. In contrast, amiRNAs are used less frequently, possibly due to their more complex design and less predictable processing, potentially resulting in less efficient silencing [125]. Nonetheless, amiRNAs designed against specific targets are just as effective as shRNAs, providing not only long-term silencing but also a higher degree of safety and fewer non-specific outcomes [126,127,128]. These features make amiRNAs promising tools for genetic therapy. Artificial miRNA can significantly enhance the application value of plants, showing excellent performance in pest and microbial resistance and even playing an important role in the field of new human medicine (e.g., Table 4).

#### 5.1.1. Artificial miRNAs in Insect Resistance

amiRNA technology holds significant potential for enhancing plant disease resistance; its application in the context of insect resistance, in particular, has been widely studied. The striped stem borer (SSB) represents a significant threat to rice (*Oryza sativa*) [129]. Nonetheless, the amiRNA csu-novel-260 inhibits the synthesis of ecdysteroids, one of the most important hormones regulating insect development [130]. Therefore, Hao et al. employed amiRNA technology to overexpress csu-novel-260 in rice, obtaining transgenic rice with significant SSB resistance [129]. Specifically, when SSB larvae consume csu-novel-260-containing transgenic rice, the suppression of ecdysteroid synthesis is induced, resulting in a high SSB mortality rate. In another study, Wen et al. overexpressed 13 small endogenous RNAs of SSB in rice using amiRNA technology; corresponding feeding assays revealed significantly inhibited SSB growth [131]. Specifically, pupation was delayed for 4 days when SSB larvae were continuously fed transgenic rice expressing the novel SSB miRNA candidate csu-novel-miR15 (csu-15). Gene expression analysis revealed a significant alteration (up- or down-regulation) in the expression levels of at least six individual SSB genes after oral consumption of csu-15 rice. Moreover, Liu et al. successfully engineered SSB-resistant rice (csu-53) expressing an artificial SSB endogenous miRNA (csu-novel-miR53) [132]. Subsequent feeding experiments demonstrated that consumption of csu-53 rice inhibits SSB larval growth, delays pupation time, and decreases pupal weight and emergence rate. In a 10-day feeding experiment, the miRNA mimic csu-novel-miR53 not only inhibited larval growth but also increased larval mortality.

Rice stem borers (RSB) cause massive annual economic losses. He et al. identified an insect-specific miRNA, miR-14, in RSB as a potential regulatory gene associated with molting [133]. Notably, csu-miR-14 is highly expressed at the end of each larval instar. Conversely, overexpression of csu-miR-14 on the third day of the fifth RSB instar results in high mortality and developmental abnormalities. Therefore, He et al. constructed transgenic rice with high miR-14 expression and observed high RSB resistance in the transgenic line.

The cotton bollworm *Helicoverpa armigera* is a major pest affecting various crops. While the expression of *Bacillus thuringiensis* (Bt) toxins in transgenic crops has successfully inhibited pests such as the cotton bollworm, field-evolved resistance has emerged in a wide range of pests. Therefore, new protective strategies must be sought to effectively suppress Bt-resistant insect populations. Pre-miRNA transcripts of insects can be modified into amiRNAs that target insect genes. Bally et al. expressed these modified genes in *Nicotiana benthamian*. The cotton bollworms that consumed transgenic *Nicotiana benthamian* leaves exhibit increased mortality, developmental abnormalities, and delayed growth [134]. Hence, expressing insect pre-amiRNAs in plants might represent a novel strategy for protecting plants from herbivorous insects. The ecdysone receptor (HaEcR) gene in the cotton bollworm is pivotal in regulating all developmental stages of the insect life cycle; nonetheless, this gene can be silenced by a sequence-specific amiRNA (amiRNA-HaEcR). Therefore, Yogindran et al. constructed transgenic tomatoes expressing amiRNA-319a-HaEcR; continuous feeding of the transgenic leaves to cotton bollworms reduced their overall growth rate and survival [135]. Alternatively, Agrawal et al. constructed a vector-producing amiR-24, specifically targeting the chitinase gene of the cotton bollworm [136]. When bollworm larvae consumed the leaves of transgenic tobacco plants with high amiR-24 expression, they exhibited molting anomalies and eventually died. Furthermore, Faisal et al. employed miRNA-mediated RNAi technology to develop transgenic tomato plants with resistance to aphids by silencing the peach aphid acetylcholinesterase 1 (*Ace1*) gene [137]. Quantitative PCR data clearly demonstrated that *Ace1* was significantly downregulated in aphids raised in clamp cages on T-1 transgenic plants. Additionally, the aphid populations reared on the T-1 transgenic plants of both tomato plant cultivars exhibited a significant reduction.

#### 5.1.2. Artificial miRNAs in Microbe Resistance

amiRNAs are widely used to investigate the resistance of plants to pathogenic microorganisms. Niu et al. constructed two amiRNA vectors by replacing the conserved miR159a backbone fragments from *A. thaliana* with the HC-Pro gene from the turnip mosaic virus (TuMV) and the P69 gene from the turnip yellow mosaic virus (TYMV). The results show that these two amiRNAs specifically silence target gene expression and are stably inherited in subsequent generations [138]. Additionally, genetic modification of tomato plants with an amiRNA vector targeting the silencing repressor 2b gene of the cucumber mosaic virus (CMV) induces effective resistance against CMV infection [139,140]. Moreover, genetic transformation of the precursor amiRNA backbones derived from different viral genes into the same plant can induce simultaneous resistance to multiple viruses. For example, Ai et al. introduced amiRNAs targeting the silencing suppressors HC-Pro (potato virus Y, PVY) and p25 (potato virus X) into *Nicotiana tabacum*. The transgenic tobacco exhibits markedly improved resistance to both diseases [141]. Similarly, Song et al. designed amiRNAs based on the PVY and tobacco etch virus (TEV) Nib and CP genes; the transgenic tobacco transfected with these amiRNAs exhibits resistance to both viruses [142].

Al-Roshdi et al. constructed amiRNAs using the miR159a hairpin structure to produce tomato plants resistant to the whitefly-transmitted tomato yellow leaf curl virus-Oman (TYLCV-OM). The transgenic tomato plants exhibit upregulated amiRNA expression, which effectively downregulates or silences the AC1/Rep transcript of TYLCV-OM, encoding enhanced tolerance against TYLCV infection [143]. Under TYLCV-OM infection conditions, viral replication is dramatically reduced in T1 transgenic tomato plants. Alternatively, Khalid et al. utilized amiRNA technology to express 14 amiRNAs targeting the conserved regions of seven TYLCLV genes and their satellite DNAs, incorporating intronic and exonic amiRNAs [144]. The resulting pAMIN14 and pAMIE14 vectors encode extensive amiRNA clusters, the silencing of which was validated by transient assays and stable transgenic *N. tabacum* plants. To assess the resistance of pAMIE14 and pAMIN14 transgenic plants to TYLCLV, the corresponding plasmids were transformed into the tomato cultivar A57; viral resistance was evaluated following mixed infection with TYLCLV. Overall, the pAMIN14 transgenic line exhibits a greater level of TYLCLV resistance than the pAMIE14 transgenic line, with resistance comparable to that of plants carrying the TY1 resistance gene. Additionally, zucchini resistant to the yellow mosaic virus (ZYMV) can be created via artificial miRNA-mediated methods [145]. These amiRNAs are derived from the gene encoding HC-Pro—a ZYMV protein involved in aphid transmission and cell-to-cell movement thought to be an RNAi repressor [146]. Hence, amiRNAs have considerable potential as crop antimicrobials.

Practical validation is required for amiRNA applications. Indeed, the transfer of amiRNA targeting potato spindle tuber viroids (PSTVd) into tomato plants causes developmental retardation and reproductive defects in the plants [147]. Furthermore, amiRNAs inadvertently silenced sterol glycosyltransferase 1 (*SISGT1*) in tomatoes, leading to impairment of the growth hormone signaling pathway and impeding the growth of amiR-PSTVd plants [148].

#### 5.1.3. Artificial miRNAs in Human-Related Disease Resistance

Plant-derived amiRNAs based on plant gene expression have been investigated for the treatment of mammalian diseases. For instance, Kakeshpour et al. constructed plant expression vectors containing amiRNAs targeting mouse complement 3 (C3) and coagulation factor 7 (CF7) mRNAs using the rice miRNA backbone Osa-miR528 [149]. The corresponding transgenic lettuce successfully expresses primary and mature amiRNAs. Subsequently, Zhang et al. produced lettuce (*Lactuca sativa* L.) expressing small silencing amiRNAs that specifically inhibit the expression of the hepatitis B virus HBsAg gene at relatively low levels compared to synthetic siRNAs, effectively alleviating liver injury in p21-HBsAg transgenic mice [150]. Continuous administration of these amiRNAs to mice maintains relatively stable amiRNA levels in vivo, markedly reducing pathological damage. While overexpression via viral transduction and exogenous substances is relatively impossible without interfering with the cell’s physiological condition, amiRNAs offer a more “natural” gene therapy tool regarding their structure, biogenesis, and expression levels. This enhances the safety of plant-derived siRNAs as RNAi therapeutics.

However, it is important to note that amiRNA technology may encounter certain challenges in agricultural applications, especially in the molecular breeding of antiviral transgenes. Moreover, all published case studies include transgenic model plants; thus, field application is not immediately available. In addition, viral genomes evolve faster than plant miRNAs, and viruses can alter the conservation of their target regions (e.g., through mutation), potentially resulting in off-target effects of the amiRNAs.

**Table 4:** Artificial miRNAs increase the value of plants.

Plant	amiRNA	Target Species		Effect	Reference(s)
Rice	csu-novel-260	Pests	Striped stem borer	Inhibits ecdysteroid synthesis	[129,130]
	csu-novel-miR15			Inhibits SSB growth	[131]
	csu-novel-miR53			Inhibits larval growth, delaying pupation time	[132]
	csu-miR-14		Rice stem borers	High mortality	[133]
Tobacco	insect pre-amiRNAs		Cotton bollworm	Increases mortality and developmental abnormalities	[134]
	amiRNA-319a-HaEcR			Reduces overall growth rate and survival	[135]
	amiR-24			Molting anomalies and death	[136]
*A. thaliana*	amiR-HC-Pro, amiR-P69	Microbe	Turnip mosaic virus	Silence target gene expression	[138]
Tomato	amiR-CMV-2b		Cucumber mosaic virus	Targets CMV infection	[139,140]
Tobacco	amiR-PVY-HC-Pro		Potato virus Y	Improves resistance	[141]
	amiR-PVX-p25		Potato virus X		
	amiR-TEV-CP		Tobacco etch virus		[142]
Tomato	amiR159a-TYLCV-OM		Tomato yellow leaf curl virus-Oman	Enhances tolerance in plants against TYLCV infection	[143]
Zucchini	amiR-HC-Pro		Yellow mosaic virus	ZYMV resistance	[145]
Tomato	amiR-PSTVd		Potato spindle tuber viroid	PSTVd resistance	[147]
Rice	Osa-miR528-C3, Osa-miR528-CF7	–	Mouse	Express primary and mature amiRNAs	[149]
Lettuce	lettuce-derived amiRNAs		Hepatitis B virus	Reduce pathological damage	[150]

### 5.2. Plant Extracellular Vesicles Can Facilitate the Cross-Species Transport of miRNAs

Extracellular vesicles (EVs) are a general term for vesicles with a membrane structure secreted by cells and represent natural nanoscale particles [151]. EVs can carry molecules such as nucleic acids, proteins, lipids, etc., and participate in specific and efficient intercellular material transfer and information exchange. EVs exist in animals, plants, and microorganisms [152]. Currently, most research on EVs focuses on mammals and those that are ~30–150 nm in size. Meanwhile, research on plants remains relatively limited. In 2009, Regente et al. discovered vesicles with a 50–200 nm diameter within the fluid collected from sunflower seeds; this represents the first known attempt to extract EVs from plants. They found that EVs can affect the normal growth and development processes of fungi, suggesting that they are involved in plant–microbial interactions [153].

To differentiate from animal EVs, plant EVs are commonly referred to as exosome-like nanovesicles (ELNVs). Although the biogenesis of ELNVs has not been fully characterized, the fusion of multi-vesicular bodies and plant-specific exocyst-positive organelles with the plasma membrane is thought to be the main source of plant ELNVs [154,155]. miRNA-rich plant ELNVs are important in plant defense against biotic stress. For example, Cai et al. reported that *A. thaliana* can inhibit gray mold virulence genes via the exosomal delivery of miRNAs and inhibit fungal infestation through a transboundary nucleic acid delivery mechanism [155].

The structure of plant-derived ELNVs is similar to that of animal-derived EVs. This provides insights regarding the potential biological functions of ELNVs, including information transmission in animal cells [156,157]. The ELNV structure can partially resist the external environment, preventing the degradation or inactivation of its contents [158]. In particular, the RNA in ELNVs remains active after oral administration [159,160,161]. A recent study evaluated the biological effects of miRNA159 loaded in ELNVs by isolating and employing four food-borne ELNVs as transport carriers [162]. Among them, the level of miRNA transfected into ELNVs is ≥20 times higher than that of untreated miRNA, and cell absorption is also relatively improved. Moreover, the delivery of ELNVs isolated from broccoli carrying miR167a effectively reduced pancreatic cancer cell activity by inhibiting IRS3 of the PI1K–AKT pathway [163]. Katia et al. further revealed the presence of miR156c and miR159a in ELNVs extracted from walnuts. These miRNAs can regulate the mammalian TNF-α signaling pathway in adipocytes, modulating inflammation; notably, they have also influenced human physiology by participating in molecular regulation in vivo [164].

This confirms the previously established hypothesis that utilizing the biogenesis and cross-kingdom delivery mechanisms of endogenous plant miRNAs not only provides valuable insights for the development of novel biopesticides but also holds promise for novel pharmaceutical applications.

### 5.3. Potential of Oral Plant miRNA in the Pharmaceutical Industry

Plant-based foods contain plant miRNAs that can enter animals and humans via oral consumption and actively participate in gene expression regulation. More specifically, dietary miRNAs can be encapsulated and released into the circulation following cellular uptake in the gastrointestinal tract; they are then transported to various tissues, similar to endogenous miRNAs [165]. These exogenous plant miRNAs subsequently participate in the regulation of gene expression, affecting physiological processes and disease development and exerting antiviral, anti-tumor, and anti-inflammatory effects.

Notably, miR2911 inhibits the replication of myriad viruses, e.g., the novel coronavirus [166], Varicella-zoster virus [167], Enterovirus 71 [168], and Influenza A viruses [166]. Zhang postulated that miR2911 is the “penicillin of virology” and suggests that it can serve as a novel therapeutic for the prevention of other viral infections [166]. In addition, miR159 is the first plant miRNA shown to inhibit tumor growth in mammals. A study conducted by Chin et al. analyzed small RNAs in the sera of 42 patients with breast cancer, revealing the presence of plant miR159 in the Western population; interestingly, higher levels of miR159 were associated with a reduced risk and progression of breast cancer [169]. Moreover, a miR156a mimic, miRNA156a, inhibits epithelial–mesenchymal transition in human nasopharyngeal cancer by targeting the 3′-UTR of junctional adhesion molecule A (JAMA) [170]. Additionally, Chen et al. demonstrated a role for plant-derived miR5338 in the treatment of benign prostatic hyperplasia in rats by inhibiting *Mfn*1 in the prostate [171].

SIDT1 is an essential factor in the transport of plant miRNAs. miRNA absorption is hypothesized to occur in the small intestine; however, the associated hostile environment poses a major challenge to the stability of orally administered plant miRNAs [172]. Hence, Zhang et al. reported that dietary miRNA uptake is conducted by the stomach, which is enriched in SIDT1 protein—a key transporter. Additionally, dietary miRNA uptake is markedly reduced following SIDT1 knockdown [173]. Subsequently, exogenous dietary miRNAs are transported into the cells via the intrinsic carrier protein SIDT1 in the stomach. These miRNAs are then secreted as functional factors in exosomes, protecting them from degradation in the bloodstream and facilitating their cellular uptake. This natural mammalian uptake pathway for dietary miRNAs can be readily exploited for the oral administration of therapeutic miRNAs, representing an important future direction for developing RNA-based therapeutics.

## 6. Discussion and Perspective

Global warming continues to expose plants to an increasing number of biological and abiotic stresses. Plant-derived miRNAs can regulate and alleviate plant stress through endogenous, cross-species, and cross-kingdom gene regulation. In this review, we have summarized the current understanding of the synthesis, self-regulation, cross-kingdom regulatory mechanisms, and potential application strategies of plant miRNAs. Overall, these plant-derived miRNAs hold potential for the research and development of novel pesticides in modern agriculture.

While a growing number of miRNAs with cross-kingdom regulatory potential have been identified, their low expression and limited utilization pose significant challenges. To promote the practical application of miRNAs in agriculture and medicine, a more rational approach to the production and modification of miRNAs is needed. In particular, this approach should take advantage of plant-specific molecular mechanisms for synthesizing and transporting miRNAs. Synthetic biology is an emerging field of biological research used to elucidate and simulate the basic laws of biosynthesis while allowing the effective artificial design and construction of novel, physiologically specific products. Synthetic biology can produce disease-specific biomarkers, acting as a powerful new tool for targeting various diseases [174]. Traditional miRNA research focuses on the genetic regulation of phenotypic traits, evaluating the regulation of mRNAs by an individual’s miRNAs. In contrast, synthetic biology is used as a bridge to combine the strengths of different species, increasing the future research potential of miRNAs.

Notably, miRNAs can be used in the cross-species regulation of plant parasites and beneficial insects [105]; this holds promise as an effective strategy for developing novel pesticides and promoting the development of modern agriculture [175]. amiRNAs can be considered species-specific insecticides, presenting a powerful alternative to traditional chemical methods [176]. These miRNAs not only support beneficial insects in eliminating diseases but can also reduce pest infestations. This can be implemented by applying pest-specific amiRNAs to the leaves and soil, disrupting key physiological pathways within pests [177]. The application of miRNAs can also be combined with existing agricultural technologies to increase crop yield and enhance crop resilience to global climate change and related agricultural challenges [178].

In this context, synthetic biology involves the isolation of miRNAs with multiple functions, such as miR2911, which exhibits resistance against multiple viruses. These miRNAs can be heterologously expressed at an industrial level using high-yield chassis cells, shifting from theory to practical application. Alternatively, a “backward-looking” search can identify target genes within specific pathogens or pests. RNAi can then be employed to express edible plant miRNAs that can specifically inhibit pathogens and pests. Subsequently, edible plant vesicles or animal-derived exosomes can be used to absorb and transport miRNAs throughout the body to specifically target pathogens and pests. As agriculture is vital worldwide, its full potential should be explored to determine the value of plant miRNAs in higher plants, particularly crops.

However, several limitations remain in the application of miRNAs in agriculture. For instance, it remains unclear whether genetically modified food can contaminate human genes following ingestion and whether genetically modified plants can have a genetic impact on surrounding organisms during cultivation. In this case, the concern might be whether an RNAi-active plant could adversely affect the consumer. Subsequently, upon ingestion, specific miRNAs can be encapsulated within ELNVs to mitigate a decrease in their biological activity; however, certain miRNAs remain exposed. Thus, some miRNAs demonstrate relative stability, such as miR2911, enabling them to traverse the digestive tract without degradation. Conversely, others experience functional loss after exposure to high temperatures and enzymatic hydrolysis. The reliable determination of systemic effects of orally administered plant-derived miRNAs remains elusive, and minimal absorption likely further restricts the possibility of sufficient miRNA quantities reaching the tissues or functional sites, limiting their potential biological impact [179]. Moreover, the breeding period of RNAi is lengthy, and the prerequisite for trait enhancement lies in non-interference with the original exceptional traits. To select and cultivate plants possessing resistance and high yields, a substantial number of field experiments are imperative. However, the modified plants can deviate significantly from expectations [148]; hence, the popularization and application warrant further verification.

## 7. Conclusions

The cross-species regulatory roles of plant miRNAs offer new prospects in agriculture, in which the understanding and application of plant miRNAs not only have the potential to enhance crop yields and resistance but also offer new insights into the development of biopesticides. Ultimately, using these miRNAs in agriculture may improve the ecological balance and reduce dependence on chemical pesticides and fertilizers. In medicine, plant miRNAs hold promise as gene therapy tools for precisely regulating specific genes. However, further research and technological development are required to address ethical, regulatory, and ecosystem impact concerns, ensuring the feasibility and sustainability of these approaches.
